# A Role for the Chemokine RANTES in Regulating CD8 T Cell Responses during Chronic Viral Infection

**DOI:** 10.1371/journal.ppat.1002098

**Published:** 2011-07-21

**Authors:** Alison Crawford, Jill Marie Angelosanto, Kim Lynn Nadwodny, Shawn D. Blackburn, E. John Wherry

**Affiliations:** 1 Department of Microbiology and Institute for Immunology, University of Pennsylvania School of Medicine, Philadelphia, Pennsylvania, United States of America; 2 GlaxoSmithKline, Department of Safety Assessment, Immunologic Toxicology, King of Prussia, Pennsylvania, United States of America; NIH/NIAID, United States of America

## Abstract

RANTES (CCL5) is a chemokine expressed by many hematopoietic and non-hematopoietic cell types that plays an important role in homing and migration of effector and memory T cells during acute infections. The RANTES receptor, CCR5, is a major target of anti-HIV drugs based on blocking viral entry. However, defects in RANTES or RANTES receptors including CCR5 can compromise immunity to acute infections in animal models and lead to more severe disease in humans infected with west Nile virus (WNV). In contrast, the role of the RANTES pathway in regulating T cell responses and immunity during chronic infection remains unclear. In this study, we demonstrate a crucial role for RANTES in the control of systemic chronic LCMV infection. In RANTES^−/−^ mice, virus-specific CD8 T cells had poor cytokine production. These RANTES^−/−^ CD8 T cells also expressed higher amounts of inhibitory receptors consistent with more severe exhaustion. Moreover, the cytotoxic ability of CD8 T cells from RANTES^−/−^ mice was reduced. Consequently, viral load was higher in the absence of RANTES. The dysfunction of T cells in the absence of RANTES was as severe as CD8 T cell responses generated in the absence of CD4 T cell help. Our results demonstrate an important role for RANTES in sustaining CD8 T cell responses during a systemic chronic viral infection.

## Author Summary

Chemokines are small proteins that attract cells and play complex roles in coordinating immune responses. RANTES is one such chemokine that attracts many different cell types. The receptor for RANTES, CCR5, is also a coreceptor for HIV and drugs blocking the RANTES∶CCR5 pathway are in clinical use to treat HIV-infected individuals. Despite the importance of CCR5 during HIV infection, the role of RANTES during other chronic infections remains poorly defined. In this study, we found that the absence of RANTES limited the ability of mice to control chronic LCMV infection resulting in higher viral loads and more severe T cell exhaustion. Our data suggest that the impact of blocking the RANTES∶CCR5 pathway on the ability to control other chronic infections should be given careful consideration when treating HIV-infected individuals.

## Introduction

During many chronic infections, virus spreads rapidly from the site of initial infection to distal tissues. T cells, on the other hand, must first become activated in the LNs and spleen and then gain the ability to migrate to infected organs. Chemokines play a key role in orchestrating all stages of this T cell response from recruitment of naïve T cells to inflamed lymphoid tissue, migration of T cells within lymphoid organs, movement of activated T cells from lymphoid tissues to effector sites, and the movement of effector T cells within non-lymphoid tissues [1]. While chemokine receptor-ligand pairs such as CCR7-CCL19/21 and CXCR5-CXCL13 are important for migration of T cells into and within lymphoid tissues, others such as CCR4-CCL17/22 and CCR10-CCL27/28 are important for T cell migration into peripheral tissues [2].

One chemokine that has been shown to play a role in immune responses to viral infections is the beta chemokine RANTES (regulated on activation normal T cell expressed and secreted). While RANTES was originally considered a T cell-specific chemokine, it is now known to be expressed by a number of other cell types including epithelial cells and platelets and acts as a potent chemoattractant for many cell types such as monocytes, NK cells [3], memory T cells [4], eosinophils [5] and DCs [6]. A receptor for RANTES, CCR5, is a G protein coupled receptor that, in addition to being the major receptor for RANTES, can also bind MIP1α (CCL3) and MIP1β (CCL4). While the importance of these and many other chemokine∶chemokine receptor pathways has been examined following acute infection or immunization, the role of specific chemokines in regulating T cell responses to chronic viral infections is less clearly defined.

One role for chemokines in regulating T cell responses is the regulation of spatial organization and cellular interactions within lymphoid tissues. For the initiation of an immune response, rare antigen-specific lymphocytes must come into contact with peptide-presenting APCs. Castellino et al showed that antigen-specific interactions of CD4 T cells with antigen-bearing DCs leads to the local production of MIP1α and MIP1β that then recruits naïve CD8 T cells to the same peptide-presenting DC activated by the CD4 T cell [7]. Thus, these chemokines can contribute to the provision of CD4 T cell help for optimal CD8 T cell priming. While Castellino et al found only a modest effect of RANTES neutralization in their protein immunization system, the relative importance of MIP-1α, MIP-1β and RANTES during infection is unknown. Given the overlap in the function of MIP-1α, MIP-1β and RANTES, these studies suggest a potential role for RANTES early in T cell responses to infection possibly via CD4 help. The importance of CD4 T cell help has long been appreciated for a number of chronic viral infections including LCMV, HCV and HIV [8], [9], [10]. When CD4 T cells are transiently depleted at the time of infection with LCMV clone 13, the mice become viremic for life in contrast to untreated mice that control viremia in 2–3 months [8]. Moreover, the CD8 T cells in the CD4 depleted mice are more severely exhausted [11]. Thus, chemokines play important roles during immune responses including aiding in the organization of tissues and in regulating cell-cell interactions.

RANTES regulates protective immunity to viral infections. For example, lymphocytes and epithelial cells produce RANTES in response to infection with respiratory syncytial virus [12] or influenza virus [13], [14], [15], [16], [17]. During respiratory infections, the RANTES∶CCR5 pathway has been shown to be important for DC migration to the dLN [18], survival of alveolar macrophages [19] and the accelerated recruitment of effector and memory T cells to the lung after challenge [20]. Evidence that chemokines can also regulate acute systemic infections arose from the infection of mice lacking CCR5 with west nile virus (WNV), which resulted in markedly higher viral titers in the central nervous system [21]. Humans with the CCR5-Δ32 genotype (a 32-base pair deletion in the CCR5 open reading frame of the CCR5 gene) also have a risk for more aggressive disease following WNV infection [22]. Thus, the RANTES∶CCR5 pathway can influence immune responses in multiple ways during acute viral infections.

In addition to the role of the RANTES∶CCR5 pathway in coordinating spatial interactions during immune responses, CCR5 is a co-receptor for HIV [23], [24]. Humans with the CCR5-Δ32 genotype have slower progression with HIV infection [25] and therapeutic strategies targeting RANTES and CCR5 are being used for treatment against HIV infection [26]. For example, the CCR5 inhibitor maraviroc, in combination with other antiretroviral agents, is indicated for patients with CCR5-tropic strains of HIV. While the benefit of maraviroc in patients with CCR5-tropic strains of HIV is clear (maraviroc can reduce viral loads), how the therapeutic targeting of the CCR5 pathway affects immune responses to other pathogens is unclear.

The role of the RANTES∶CCR5 pathway in respiratory infections, WNV infection and HIV infection suggests that the function of this pathway could be important during other viral infections and that the effect of RANTES during HIV infection might be complex. For example, the CCR5-Δ32 is beneficial during HIV infection because of a direct impediment to viral entry, however, this same mutation is detrimental during WNV infection [27] as well as tick-borne encephalitis [28]. Subjects with the CCR5-Δ32 mutation also have reduced DTH responses [25]. In contrast to acute infections with WNV, influenza virus and Sendai virus, little information exists on how RANTES impacts the T cell function or control of chronic viral infection where viral entry is not affected by CCR5 or RANTES. Thus, we used the mouse model of acute or chronic LCMV infection to investigate the role of RANTES in sustaining CD8 T cell responses during chronic infection. RANTES expression is upregulated during acute LCMV infection [29], [30] but very little is known about the expression or role of RANTES during chronic LCMV infection. Here we demonstrate that RANTES is upregulated to a much higher degree during chronic LCMV infection compared to acute LCMV infection. Unlike acute LCMV infection where RANTES deficiency had little impact on T cell responses or viral control, the absence of RANTES during chronic LCMV infection led to more severe CD8 T cell exhaustion including compromised cytokine production, higher inhibitory receptor expression and reduced cytotoxicity. The loss of IFNγ production coincided with a decrease in Tbet expression similar to levels seen in CD4-depleted mice during chronic LCMV infection. This increase in T cell dysfunction in the absence of RANTES corresponded to a substantially reduced ability to control chronic infection compared to WT mice but was not due to an intrinsic requirement of CD8 T cells to produce or respond to RANTES directly. These results suggest that manipulation of the RANTES pathway may hinder immune responses to, and thus control of, chronic infection with some pathogens.

## Materials and Methods

### Mice

C57BL/6 and Ly5.1 mice were purchased from the National Cancer Institute (NCI). CCR5^−/−^ mice were purchased from Jackson laboratories (Bar Harbor, Maine). RANTES^−/−^ mice were a gift from Michael Holtzman, Washington University St Louis and bred in-house at AALAC-approved animal care facility at the Wistar Institute, Philadelphia, PA. P14 mice were maintained at the Wistar Institute and crossed to the RANTES^−/−^ mice.

### Viruses

For primary infections, mice were infected with either LCMV Armstrong (2×10^5^ pfu) i.p. or LCMV clone 13 (2×10^6^ pfu) i.v. For re-infections, mice were infected intranasally (i.n.) with recombinant influenza virus expressing the LCMV GP33 epitope (x31-GP33, 1.6×10^5^ TCID_50_). Prior to i.n. infection, mice were anaesthetized by intraperitoneal injection of ketamine hydrochloride and xylazine (Phoenix Scientific) in 0.2 ml of PBS. Recombinant influenza strains were obtained from Dr. Richard J. Webby and were propagated in specific-pathogen-free eggs and stored at −80°C before use.

### Adoptive transfer

For adoptive transfer experiments, single-cell suspensions of CD8 T cells were equalized for the number of antigen-specific CD8 T cells and adoptively transferred by i.v. injection into the tail vein. CD8 T cells were purified (>90% purity) from whole lymphocytes using magnetic beads (CD8^+^ T cell isolation kit, MACS beads; Miltenyi Biotec) and the CD8 T cells stained with tetramer and the numbers of LCMV-specific CD8 T cells normalized before being transferred i.v. For the P14 experiments, LNs were isolated from P14 WT or P14 RANTES^−/−^ mice. The number of P14 cells was equalized and a total of 1,000 P14 cells were transferred into C57BL/6 mice at a 50∶50 ratio. Mice were infected the following day with LCMV clone 13.

### Bone-marrow chimeras

Ly5.1 mice from NCI were irradiated with 950 RADS. The following day, bone-marrow cells from Ly5.1 WT mice and Ly5.2 RANTES^−/−^ mice or Ly.2 CCR5^−/−^ mice were depleted of T, B and NK cells with MACs magnetic beads and adoptively transferred i.v. at a 1∶1 ratio. A total of 1–5×10^6^ BM cells were transferred per mouse. Mice were fed antibiotics for 2 weeks following irradiation and allowed to reconstitute for eight weeks before use.

### Isolation of lymphocytes from tissues

Mice were euthanized and the hepatic vein cut. The liver was perfused by injecting PBS into the left heart ventricle. Livers were incubated in 0.25 mg/ml collagenase D (Roche Diagnostics) and 1 U/ml DNase I (Roche Diagnostics) at 37°C for 30 min. Digested livers were homogenized using a cell strainer, applied to a 44/56% Percoll gradient, centrifuged at 850 g for 20 mins at 4°C and the lymphocyte population was harvested from the interface. Red blood cells were lysed using ACK lysing buffer (Quality Biological) before cells were washed and counted. Spleens were homogenized using a cell strainer. Red blood cells were lysed using ACK lysing buffer and the cells washed and counted.

### Flow cytometry and intracellular cytokine staining

Lymphocytes isolated from different tissues were stained using standard techniques and analyzed by flow cytometry. Virus-specific CD4 and CD8 T cells were analyzed at the peak of the response (LCMV day 8) and in the memory/chronic phase (>day 30). Virus-specific T cells were quantified in tissues using MHC-I and MHC-II tetramer staining. MHC class I peptide tetramers were made and used as described [31]. MHC-II tetramer was obtained from the NIH Tetramer Core Facility (Emory University, Atlanta, GA). For examination of cytokine production, 1×10^6^ splenocytes were cultured in the absence or presence of the indicated peptide (0.2 µg/ml for CD8 peptides and 2 µg/ml for GP66-77) and brefeldin A for 5 h at 37°C. Intracellular cytokine staining was carried out using the BD cytofix/cytoperm kit followed by antibodies for IFNγ, TNFα, IL-2 and MIP-1α. Samples were collected using the LSR II flow cytometer (Becton Dickinson). For CD107a staining, the antibody was added during the stimulation as described [32].

### RANTES ELISA

Activated CD8 and CD4 T cells were sorted using a FACSAria (BD Biosciences). Cells were stimulated with PMA/ionomycin for five hours and the supernatant used for ELISAs. The RANTES ELISA was purchased from Peprotech (Rocky Hill, NJ) and carried out according to the manufacturer's instructions.

### RT-PCR

D^b^GP33-specific CD8 T cells and IA^b^GP66-specific CD4 T cells were sorted on a FACSAria (BD Biosciences). RNA extraction was performed with Trizol (Invitrogen). cDNA was generated using the High Capacity cDNA Archive Kit (Applied Biosystems). Relative quantification real-time PCR was performed on an ABI Prism 7000 with primers purchased from Applied Biosystems. HPRT was used as an endogenous control. Results are expressed relative to naïve cells.

### Luminex assay

C57Bl/6 mice were infected with LCMV Armstrong or clone 13 and bled at day 8 and day 32 p.i. Serum samples were sent to Glaxo Smithkline for examination of RANTES protein by the luminex assay.

### In vitro cytotoxicity assay

Protocol was similar to [33]. Ly5.1^+^ splenocytes were labeled with carboxyfluorescein diacetate succinimidyl ester (CFSE); half with 100 nM CFSE and half with 1.25 µM CFSE. The CFSE-labeled cells were then pulsed with 2 µg/ml of GP33-44 or OVA257-264 peptide, respectively, for 90 mins at 37°C and then rinsed three times in RPMI with 10% fetal calf serum. The peptide pulsed targets were incubated with magnetic bead purified Ly5.2^+^ CD8^+^ T cells from spleens of WT or RANTES^−/−^ mice with a 2∶1 effector∶target ratio for 18 h. Cells were washed and stained with Ly5.1 and Live/Dead fixable red dead cell stain kit from Invitrogen (Carlsbad, CA). The killing efficiency was determined as previously described [33].

### Statistical analysis

Data were analyzed using a two-tailed Student's t-test and a p value of ≤0.05 was considered significant.

### Ethics statement

All animal experiments were performed in accordance to NIH guidelines, the Animal Welfare Act, and US federal law. The experiments were approved by the Wistar Institutes Institutional Animal Care and Use (IACUC) committee, animal welfare assurance number A3432-01. The Wistar Animal Care and Use Program is fully accredited by the Association for Assessment and Accreditation of Laboratory Animal Care International (AAALAC).

## Results

### Antiviral T cell responses are similar in RANTES^−/−^ and WT mice during acute LCMV infection

**Figure 1:**
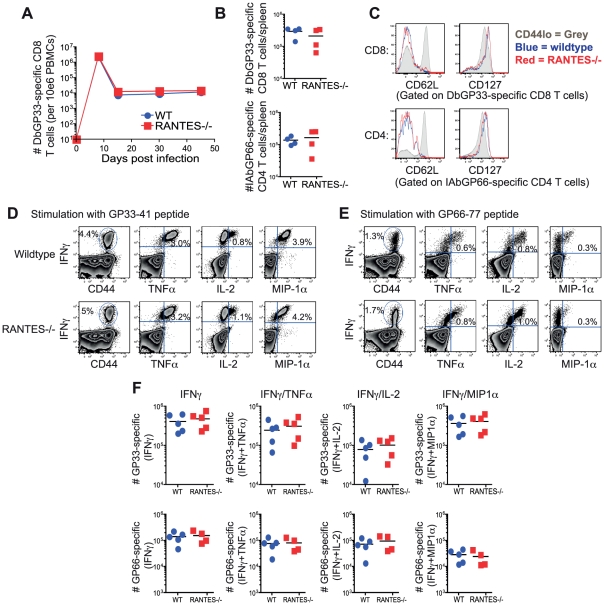
The absence of RANTES does not affect T cell responses to acute LCMV infection. WT and RANTES^−/−^ mice were infected with LCMV Armstrong. Mice were bled on days 8, 15, 30 and 45 p.i. and T cell responses examined. (**A**) The frequency of D^b^GP33-specific CD8 T cells in the blood in WT and RANTES^−/−^ mice was determined. Splenocytes from WT and RANTES^−/−^ mice were examined 52 days p.i. (**B–F**). Total numbers of D^b^GP33-specific CD8 and IA^b^GP66-specific CD4 T cells in the spleen at the memory phase of the response were determined using tetramers (**B**). CD62L and CD127 expression was examined on LCMV-specific memory CD4 and CD8 T cells from both WT and RANTES^−/−^ mice (**C**). Splenocytes from WT and RANTES^−/−^ mice were stimulated with GP33 and GP66 peptides to measure cytokine responses from CD8 and CD4 T cells, respectively (**D–F**). The cytokines IFNγ, TNFα, IL-2 and MIP-1α were measured and representative FACs plots are shown. Graphs show total numbers per spleen. Data are representative of 2 independent experiments with at least 4 mice per group in each experiment.

Infection of mice with the Armstrong strain of LCMV results in an acute infection that is cleared within 8–10 days. CD8 T cells are important for the control of acute LCMV infection and competent CD4 T cell help is required for optimal memory CD8 T cells to develop [34], [35], [36]. We infected both WT and RANTES^−/−^ mice with LCMV Armstrong to determine whether RANTES played a role in regulating T cell responses to this infection. WT and RANTES^−/−^ mice were equally capable of clearing infection with LCMV Armstrong (data not shown). LCMV-specific CD8 T cells expanded similarly in the blood and resulted in comparable absolute numbers of antiviral memory CD4 and CD8 T cells (**figure 1A and B**). Moreover, the expression of CD62L and CD127 on virus-specific memory T cells on day 52 p.i. was similar in the presence or absence of RANTES (**figure 1C**) suggesting that the pattern of memory T cell differentiation was unchanged in the absence of this chemokine. Virus-specific memory CD4 and CD8 T cells from RANTES^−/−^ mice were also able to co-produce multiple cytokines equally well (**figure 1D, E and F**) again showing that there was little, if any, influence of RANTES deficiency on the pattern of differentiation of anti-viral CD4 and CD8 T cell responses during acute LCMV infection.

### Memory CD8 T cells from RANTES^−/−^ mice generate efficient secondary responses

**Figure 2:**
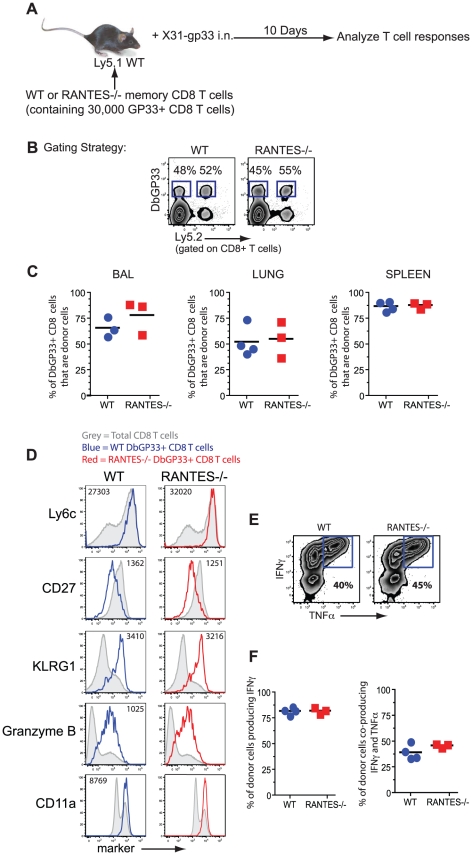
WT and RANTES^−/−^ mice mount equivalent secondary responses. (**A**) WT and RANTES^−/−^ mice were infected with LCMV Armstrong. After ∼50 days, CD8 T cells were purified from the mice and equal numbers of WT or RANTES^−/−^ D^b^GP33-specific CD8 T cells were transferred into congenically marked Ly5.1 mice. The mice were then infected the following day with X31-gp33 i.n. Ten days later, the number of donor (Ly5.2+) virus-specific CD8 T cells was enumerated by tetramer staining. Gating strategy to identify donor responses (**B**). WT and RANTES^−/−^ memory T cells were measured in the BAL (bronchoalveolar lavage), lung and spleen of recipient mice (**C**). WT and RANTES^−/−^ D^b^GP33-specific CD8 T cells were examined in the spleen and stained for Ly6c, CD27, KLRG1, granzyme B and CD11a (**D**). Production of IFNγ or TNFα by the adoptively transferred cells was measured in the spleen by peptide stimulation and ICS (**E and F**). Data are representative of 2 independent experiments each with 3 mice per group.

Given the role of the beta chemokines in regulating ‘helped’ CD8 T cell memory [7], we tested whether the memory CD8 T cells formed during acute LCMV infection could generate an anamnestic response, a key feature of optimal memory CD8 T cells. WT or RANTES^−/−^ mice were infected with LCMV Armstrong to generate GP33-specific memory CD8 T cells. CD8 T cells were isolated from WT and RANTES^−/−^ mice on day 52 p.i and equal numbers of D^b^GP33-specific CD8 T cells were adoptively transferred to congenically marked WT recipient mice. These recipient mice were then infected intranasally with influenza virus expressing the LCMV GP33 epitope (**figure 2A**). The ability of donor WT or RANTES^−/−^ memory GP33-specific CD8 T cells to expand upon rechallenge was assessed on day 10 p.i. Both WT and RANTES^−/−^ GP33-specific CD8 T cells expanded vigorously and to a similar degree (**figure 2C**). Moreover, the RANTES^−/−^ memory cells formed secondary effector CD8 T cells that were phenotypically and functionally similar to WT secondary effectors (**figure 2D–F**). Thus, memory CD8 T cells generated in the absence of RANTES were fully functional, responded efficiently to local infection rechallenge and showed evidence of having received CD4 T cell help during priming.

### Memory CD8 T cells generated in the absence of RANTES can protect from LCMV clone 13 infection

**Figure 3:**
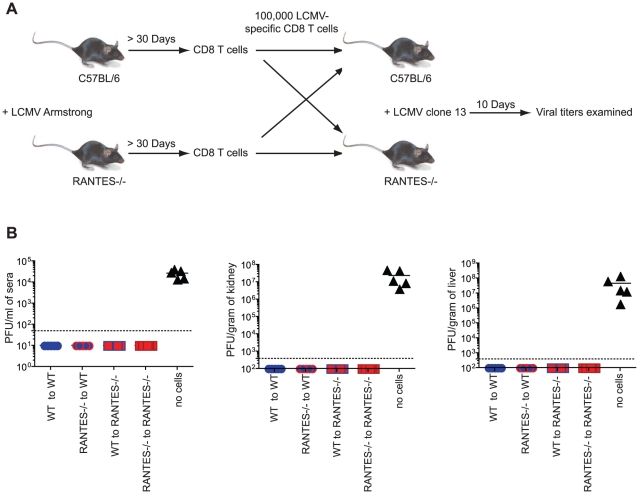
Memory CD8 T cells do not need RANTES to protect from chronic LCMV infection. (**A**) WT and RANTES^−/−^ mice were infected with LCMV Armstrong. Over 30 days later, CD8 T cells were purified from the mice and 100,000 WT or RANTES^−/−^ D^b^GP33-specific CD8 T cells were adoptively transferred into WT or RANTES^−/−^ mice (**A**). The mice were then infected the following day with LCMV clone 13 i.v. Nine days later, viral titers were measured in the spleen, kidney and sera of these mice (**B**). The results of two independent experiments shown in the same graph.

Memory CD8 T cells generated in response to LCMV Armstrong are able to protect from infection with the more virulent strain LCMV clone 13. To determine whether memory CD8 T cells generated in the absence of RANTES were able to protect from LCMV clone 13 infection, we adoptively transferred equal numbers of either WT or RANTES^−/−^ memory CD8 T cells into naïve WT or RANTES^−/−^ mice and then challenged with LCMV clone 13. As a control, a cohort of WT mice did not receive any cells. After 9 days, the mice were sacrificed and the viral loads examined (**figure 3a**). The mice that did not receive any cells had high viral titers in the serum and kidneys (**figure 3b**). In contrast, both WT and RANTES^−/−^ mice that received either WT or RANTES^−/−^ memory CD8 T cells were protected against chronic infection. Thus, RANTES was not required for memory CD8 T cells to protect from LCMV clone 13 infection.

### RANTES is highly expressed during chronic LCMV infection

Infection of naïve adult mice with LCMV clone 13 results in a chronic infection with viremia lasting 2–3 months. In contrast to LCMV Armstrong infection, during clone 13 infection the virus-specific CD8 T cells lose the ability to perform effector functions efficiently. This “exhaustion” is hierarchical and progressive with virus-specific CD8 T cells gradually losing the ability to produce IL-2, proliferate robustly, kill efficiently, make TNFα and, in severe exhaustion, produce IFNγ [31]. These exhausted CD8 T cells also express inhibitory receptors such as PD-1, LAG-3, 2B4 and CD160 [37], [38]. These receptors are actively involved in restraining CD8 T cell function during chronic infection and blockade of these pathways can reinvigorate antiviral T cell responses [32], [38].

**Figure 4:**
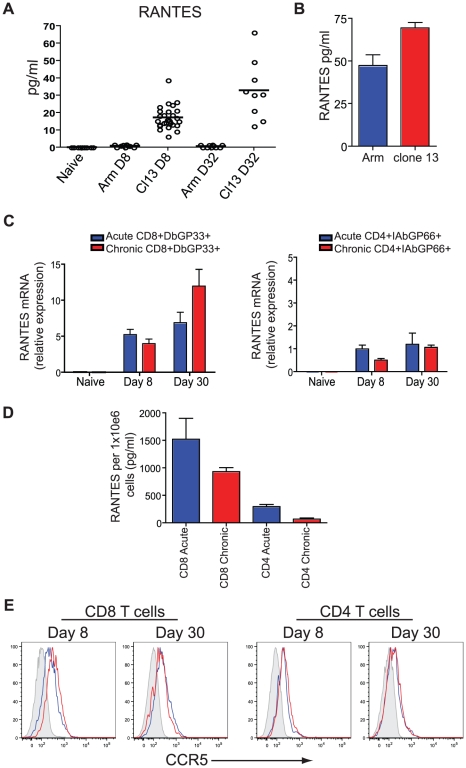
Higher concentrations of RANTES protein are present in the serum of mice infected with LCMV clone 13 compared to LCMV Armstrong and naïve mice. (**A**) C57Bl/6 mice were infected with LCMV Armstrong or LCMV clone 13 and the sera examined 8 and 32 days later for RANTES protein using luminex. (**B**) C57Bl/6 mice were infected with LCMV Armstrong or LCMV clone 13 and the sera examined 6 days later for RANTES protein by ELISA. A total of three mice each were infected. Data are representative of 2 independent experiments. (**C**) Naïve CD8 T cells and D^b^GP33-specific CD8 T cells from day 8 and day 30 p.i. with LCMV Armstrong or clone 13 were examined for expression of RANTES mRNA by RT-PCR. (**D**) Mice were infected with LCMV Armstrong or clone 13 and CD44^hi^ CD4 and CD8 T cells sorted on day 8 p.i. Sorted CD8 T cells were incubated with PMA/ionomycin for 5 hours and the supernatants examined for RANTES protein. (**E**) C57Bl/6 mice were infected with LCMV Armstrong or LCMV clone 13 and 8 and 30 days p.i. D^b^GP33-specific CD8 T cells were examined for expression of CCR5 (grey = naïve, blue = LCMV Armstrong, red = LCMV clone 13.

To begin to address the role of RANTES during chronic infection we first measured RANTES protein in serum. During LCMV clone 13 infection, RANTES levels are increased in the serum at day 8 and day 32 p.i. compared to naïve mice and mice infected with LCMV Armstrong (**figure 4A**). RANTES expression was also examined at day 6 p.i., when virus was still present in both sets of mice. Both LCMV Armstrong and LCMV clone 13 induced RANTES expression early p.i. (**figure 4B** and [39] [30]) but high amounts of circulating RANTES were sustained only during LCMV clone 13 infection. Both LCMV-specific CD8 T cells and CD4 T cells upregulated RANTES mRNA expression, with a high amount of RANTES mRNA maintained in LCMV-specific CD8 T cells past day 30 following LCMV Armstrong or clone 13 infection (**figure 4C**). Given that RANTES transcription can continue in the absence of protein production [40] and that RANTES protein can be stored in granules in the absence of secretion [41], we also measured secreted RANTES protein. CD8^+^CD44^hi^ T cells and CD4^+^CD44^hi^ T cells were sorted from mice infected eight days previously with LCMV Armstrong or LCMV clone 13 and RANTES secretion measured after 5 hours of stimulation with PMA/ionomycin. CD8 T cells from LCMV Armstrong- or clone 13-infected mice secreted high levels of RANTES protein following stimulation with PMA/ionomycin (**figure 4D**). CD4 T cells also secreted RANTES, though the amounts were lower compared to CD8 T cells (**figure 4D**). Thus, while the high amounts of circulating RANTES found in mice with chronic LCMV infection could come from many cell types, T cells clearly have the potential to contribute to this circulating chemokine production particularly in the presence of persisting antigen. Expression of the main receptor for RANTES, CCR5, is also upregulated on LCMV-specific CD4 and CD8 T cells during both acute and chronic LCMV infection suggesting that not only do T cells produce RANTES upon infection but they also have an increased ability to bind RANTES (**figure 4E**).

### CD8 T cell function is reduced during chronic LCMV infection in the absence of RANTES

**Figure 5:**
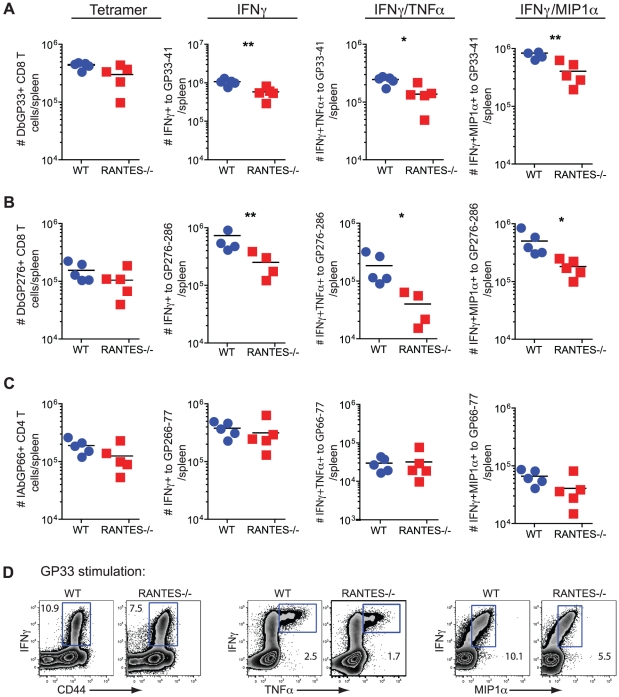
The primary CD8 T cell cytokine response is diminished in the absence of RANTES at one week after clone 13 infection. WT and RANTES^−/−^ mice were infected with LCMV clone 13 and T cell responses examined eight days later. The total number of LCMV-specific CD8 and CD4 T cells was measured by tetramer staining as well as peptide stimulation and ICS to detect cytokine production. Expression of IFNγ, TNFα and MIP1α was examined (**A–D**). Graphs show total numbers per spleen. Representative plots of IFNγ, TNFα and MIP-1α expression (**D**). Data are representative of 3 independent experiments with five mice per group.

Given the high circulating amounts of RANTES during LCMV clone 13 infection we next investigated whether RANTES had any role during chronic infection. WT and RANTES^−/−^ mice were infected with LCMV clone 13 and T cell responses examined eight days later. While the total number of D^b^GP33 and D^b^GP276 tetramer positive CD8 T cells as well as IA^b^GP66 tetramer specific CD4 T cells were similar in WT and RANTES^−/−^ mice, the total number of GP33- and GP276-specific CD8 T cells producing IFNγ was significantly reduced in RANTES^−/−^ mice (**figure 5A and B**). This difference in functionality was only observed in virus-specific CD8 T cells but not CD4 T cells as GP66-specific CD4 T cells from WT and RANTES^−/−^ mice had similar cytokine co-production profiles (**figure 5B, C and D**). Thus, CD8 T cell responses (but not CD4 responses) are functionally compromised at day 8 p.i. in the absence of RANTES during LCMV clone 13 infection.

**Figure 6:**
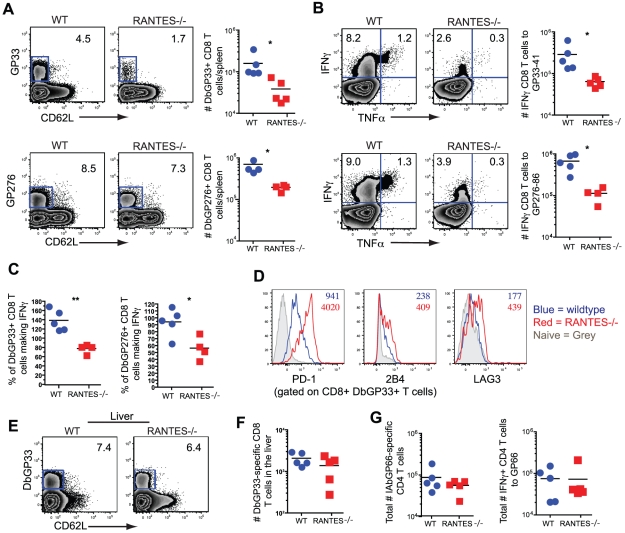
CD8 T cell responses are significantly reduced in RANTES^−/−^mice one month p.i. The total numbers of LCMV-specific CD8 T cells were examined in WT and RANTES^−/−^ mice 30 days p.i. with LCMV clone 13. Representative FACs plots are shown on the left (**A**). The ability to produce IFNγ was measured by peptide stimulation and ICS (**B**). The percentage of LCMV tetramer +ve cells able to make IFNγ was calculated from the total number of tetramer +ve CD8 T cells and total number of IFNγ-producing T cells in response to the same peptide (**C**). D^b^GP33-specific CD8 T cells from WT and RANTES^−/−^ mice were stained for PD-1, 2B4 and LAG3. Representative plots are shown. Numbers represent the MFI. Grey = naïve, blue = D^b^GP33-specific CD8 T cells from acute infection, red = D^b^GP33-specific CD8 T cells from chronic infection (**D**). D^b^GP33-specific CD8 T cells were examined in the liver. Representative plots with numbers indicating the percentage of CD8 T cells that were D^b^GP33-specific (**E**). Total numbers of D^b^GP33-specific CD8 T cells in the liver were determined by tetramer staining. (**F**). The total number of IA^b^GP66-speciifc CD4 T cells were determined by tetramer staining as well as peptide stimulation and ICS (**G**). Data are representative of three independent experiments each with at least four mice per group.

To determine whether the absence of RANTES led to a change in the development of T cell exhaustion, we examined later timepoints during clone 13 infection. In contrast to day 8 p.i., at day 30 p.i. the number of virus-specific CD8 T cells in the RANTES^−/−^ mice determined by tetramer staining was significantly reduced compared to WT mice (**figure 6A**). The reduced LCMV-specific CD8 T cell responses in the spleen were unlikely to be due to enhanced migration to peripheral tissues since the LCMV-specific CD8 T cell response was not increased in the liver (**figure 6E and F**). Even though both WT and RANTES^−/−^ CD8 T cells were highly dysfunctional at this time, exhaustion was substantially more severe in the absence of RANTES (**figure 6B and 6C**). Indeed, LCMV GP33- and GP276-specific CD8 T cells were significantly less polyfunctional (i.e. more exhausted) in RANTES^−/−^ compared to WT mice (**figure 6B**) suggesting that the absence of RANTES led to more severe exhaustion of virus-specific CD8 T cells. Similar to day 8 p.i., the LCMV-specific CD4 T cell response was unaffected by the absence of RANTES in terms of numbers of tetramer-specific CD4 T cells and production of IFNγ (**figure 6G**). A second hallmark of T cell exhaustion is elevated expression of inhibitory receptors. RANTES^−/−^ virus-specific CD8 T cells had higher expression of PD1, LAG3 and 2B4 indicating that by multiple parameters virus-specific CD8 T cells are more exhausted in the absence of RANTES (**figure 6D**).

**Figure 7:**
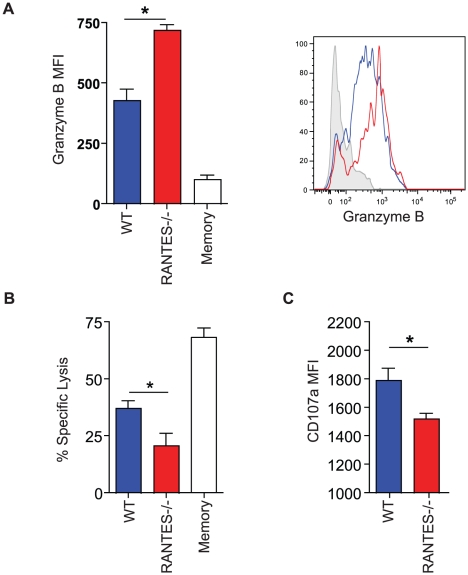
The cytotoxic ability of virus-specific CD8 T cells was decreased in the absence of RANTES. WT and RANTES^−/−^ mice were infected with LCMV clone 13 and 30 days later. LCMV-specific CD8 T cells were examined for expression of granzyme B (**A**). Results are shown graphically (left) and a representative histogram is shown. Grey = Naïve CD8 T cells, Blue = D^b^GP33-specific CD8 T cells from acute infection, red = D^b^GP33-specific CD8 T cells from chronic infection. Equal number of GP33-specific CD8 T cells were examined for their ability to kill CFSE-labeled target cells (**B**). Surface CD107a was measured during a 5 hour stimulation (**C**).

The cytotoxic ability of CD8 T cells is critical during chronic infections. While granzyme B levels were slightly higher in LCMV-specific CD8 T cells from RANTES^−/−^ mice (**figure 7A**), the ability of these cells to kill was lower than WT T cells from chronically infected mice (**figure 7B**). Degranulation, as measured by surface CD107a staining, was also slightly lower in LCMV-specific CD8 T cells from RANTES^−/−^ mice suggesting that granule contents might not be released as effectively by CD8 T cells from RANTES^−/−^ mice leading to an accumulation of granzyme B intracellularly (**figure 7C**).

**Figure 8:**
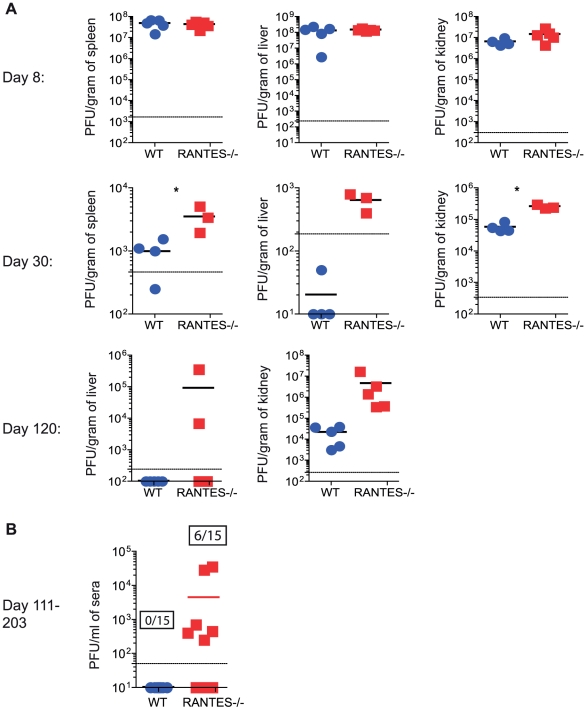
Higher viral loads later during chronic LCMV infection in RANTES^−/−^ versus WT mice. WT and RANTES^−/−^ mice were infected with LCMV clone 13 and viral titers were determined by plaque assay from tissues at 8, 30 and 102 days p.i. (**A**). Sera was also examined on days 111–203 p.i. The graph shows the result of three independent experiments shown together (**B**). Ratios show the number of mice that were viremic in the WT and RANTES^−/−^ groups out of a total of 15 mice.

Given the reduced cytokine production and cytotoxicity in CD8 T cells from mice lacking RANTES, we examined whether these T cell defects had an impact on viral control. At day 8 p.i., viral titers in RANTES^−/−^ mice were similar to WT mice in multiple tissues and sera (**figure 8A**). However, by day 30 p.i., RANTES^−/−^ mice had higher viral load, consistent with a reduced and more dysfunctional CD8 T cell response (**figure 8A**). Moreover, when RANTES^−/−^ mice were examined 3–4 months p.i., some of the RANTES^−/−^ mice still had high levels of virus in the liver and were still viremic (**figure 8A and B**) while WT mice had controlled virus from the serum. These results demonstrate that the absence of RANTES compromises the ability to control viral replication, in some cases leading to a long-term failure to efficiently contain persisting infection.

### Providing RANTES in trans is sufficient for normal CD8 T cell responses to chronic infection

**Figure 9:**
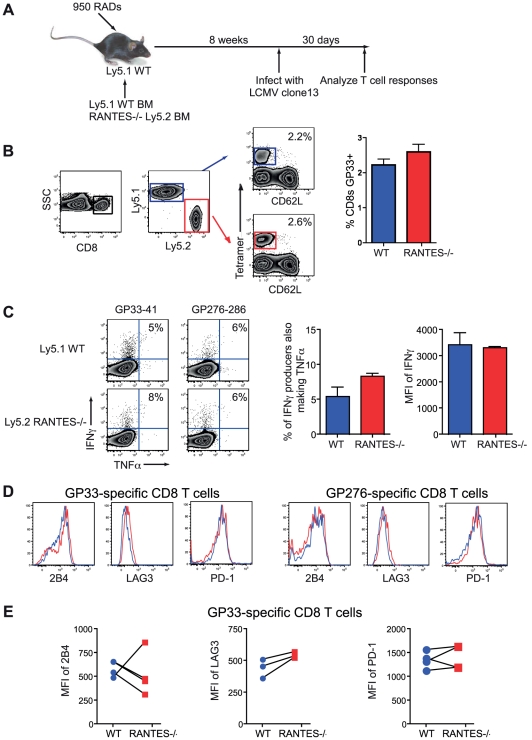
CD8 T cells do not need to produce RANTES themselves. Mixed bone-marrow chimeras were made where ∼50% of the cells were WT Ly5.1+ and ∼50% RANTES^−/−^ Ly5.2+ (**A**). Upon reconstitution, mice were infected with LCMV clone 13 and the CD8 T cell responses analyzed. RANTES^−/−^ T cells were identified by staining with Ly5.2 (**B**). Both the RANTES^−/−^ and WT CD8 T cells were examined for their ability to produce IFNγ and TNFα (**C**). Representative FACs plots are shown (left) as well as a bar graph summarizing the percentage of IFNγ-producers able to make TNFα and a graph of the MFI of IFNγ. Both WT and RANTES^−/−^ T cells were stained for 2B4, LAG3 and PD-1 with (**D**) showing representative plots of staining. Graphs plot the MFI of 2B4, LAG3 and PD-1 (**E**).

Given the reduction in CD8 T cell responses and greater exhaustion in RANTES^−/−^ mice we made mixed bone-marrow chimeras to determine whether the role of RANTES was T cell intrinsic or whether supplying RANTES in trans could prevent more severe CD8 T cell dysfunction. Congenically marked Ly5.1 mice were lethally irradiated and reconstituted with 50% Ly5.1 BM and 50% Ly5.2 RANTES^−/−^ BM (**figure 9A**). These mice were infected with LCMV clone 13 and examined thirty days later. LCMV-specific CD8 T cell responses were similar for WT versus RANTES^−/−^ CD8 T cells at this time point (**figure 9B**). Moreover, in a situation where ∼ half of the cells were able to produce RANTES, the RANTES^−/−^ CD8 T cells were as functional as WT T cells in terms of the percentage of IFNγ-producers able to make TNFα and the MFI of IFNγ (right) (**figure 9C**). Finally, RANTES^−/−^ T cells in the mixed chimeras had similar expression of the inhibitory receptors 2B4, PD-1 and LAG-3 as WT T cells (**Figure 9D, E**). We also used a non-bone marrow chimera TCR transgenic adoptive transfer system to determine whether the need for RANTES was T cell intrinsic. P14 mice bearing a T cell receptor specific for the D^b^GP33 epitope from LCMV were crossed to RANTES^−/−^ mice. Equal numbers of WT and RANTES^−/−^ P14 CD8 T cells were co-transferred into WT mice before infection with LCMV clone 13 and the CD8 T cell responses examined. Again, LCMV-specific CD8 T cells did not need to make RANTES themselves since the expression of PD-1 and the ability to make IFNγ and TNFα was similar between WT and RANTES^−/−^ P14 cells in the same chronically infected mice (**figure S1**). Thus, the critical role of RANTES in sustaining T cell responses during chronic LCMV infection was not cell intrinsic. In other words, the defects in T cell responses to chronic viral infections observed in the complete absence of RANTES could be corrected by providing RANTES signals in trans.

### CD8 T cells do not need intrinsic CCR5 signaling to respond to chronic LCMV infection

**Figure 10:**
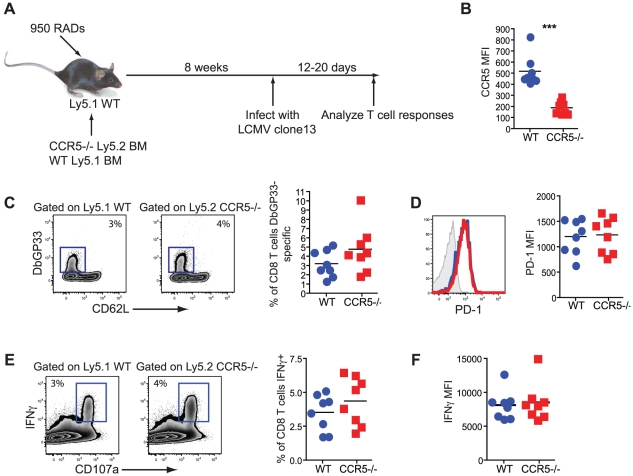
CD8 T cells do not need to bind RANTES directly. Mixed bone-marrow chimeras were made where ∼50% of the cells were Ly5.1+ WT and ∼50% Ly5.2+ CCR5^−/−^. Upon reconstitution, mice were infected with LCMV clone 13 and the CD8 T cell responses analyzed 12–20 days later (**A**). The WT and CCR5^−/−^ IA^b^GP33-specific CD8 T cells were stained for CCR5 (**B**). The percentage of WT and CCR5^−/−^ CD8 T cells that were GP33-specific were determined by D^b^GP33 tetramer (**C**). Representative staining of PD-1 on D^b^GP33-specific CD8 T cells and a graph of the MFI is shown (**D**). IFNγ production was measured in response to GP33-44 peptide stimulation (**E**). Representative FACs plots are shown (left) as well as graphs showing the percentage of the WT or CCR5^−/−^ CD8 T cells producing IFNγ and the MFI of IFNγ (**F**). Data are representative of 2 independent experiments each with at least 5 mice per group.

While intrinsic RANTES production was not required by the CD8 T cells, it remained possible that the CD8 T cells need to bind RANTES themselves. To test this idea we generated mixed bone marrow chimeras using Ly5.1 WT and Ly5.2 CCR5^−/−^ BM (**figure 10A**). Upon reconstitution, mice were infected with LCMV clone 13 and CD8 T cell responses examined. This chimera system confirmed that WT LCMV-specific CD8 T cells expressed CCR5 during chronic LCMV infection (**figure 10B**). A similar response was observed for WT and CCR5^−/−^ CD8 T cells in this setting as measured by the frequency of D^b^GP33 positive CD8 T cells (**figure 10C**). Expression of PD-1 and production of IFNγ was also similar for WT and CCR5^−/−^ LCMV-specific CD8 T cells (**figure 10D, E and F**). Thus, it appears that the major impact of RANTES during chronic LCMV infection could be on a non-CD8 T cell and that more severe CD8 T cell exhaustion was a symptom rather than a cause of poor control of infection.

### Absence of CD4 T cell help or RANTES results in reduced Tbet expression during chronic infection

**Figure 11:**
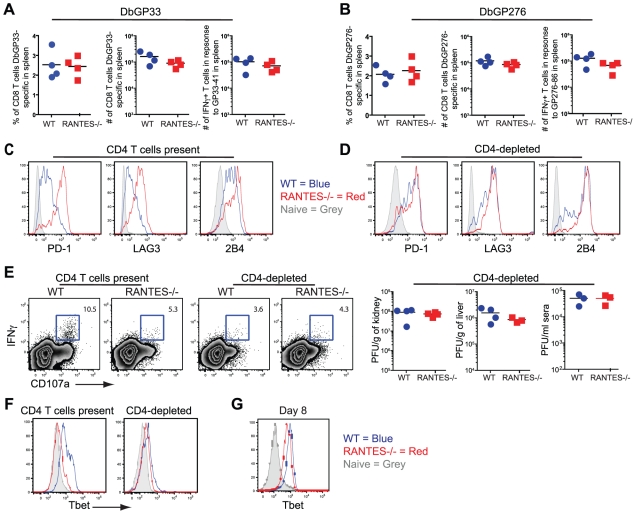
CD4-depletion reduces Tbet and IFNγ production in WT mice similar to levels seen in RANTES^−/−^ mice. A cohort of WT and RANTES^−/−^ mice were depleted of CD4 T cells with GK1.5 antibody on the day prior to infection with LCMV clone 13. T cell responses were examined 35 days later. (**A and B**) Percentages and total numbers of LCMV-specific CD8 T cells were determined in WT and RANTES^−/−^ mice depleted of CD4 T cells for both D^b^GP33-specific and D^b^GP276-specific CD8 T cells using tetramer. (**C**) D^b^GP33 and D^b^GP276-specific CD8 T cells were examined for expression of PD1, LAG3 and 2B4. Filled grey represents naïve CD8 T cells, blue = DbGP33-specific CD8 T cells from WT mice, red = DbGP33-specific CD8 T cells from RANTES^−/−^ mice. (**D**) Representative plots of CD107a expression and IFNγ production by D^b^GP33-specific CD8 T cells. Numbers represent the percent of CD107a+ cells that are also making IFNγ in response to stimulation with GP33-44 peptide. Viral titers were determined by plaque assays in CD4-depleted WT and RANTES^−/−^ mice (**E**). Tbet expression was determined in D^b^GP33-specific CD8 T cells by flow cytometry in WT and RANTES^−/−^ mice containing CD4 T cells as well as those depleted of T cells prior to infection (**F**). Tbet expression was already slightly reduced by 8 days p.i. (**G**).

The transient depletion of CD4 T cells at the time of infection with LCMV clone 13 results in life-long viremia and high viral titers throughout the mouse [8]. This deficiency coincides with more severe exhaustion of the CD8 T cell response demonstrated by further diminished cytokine production [31], [42]. Given the reduced cytokine potential of RANTES^−/−^ mice, we examined how this dysfunction compared to CD4-depleted WT mice and whether CD4 T cell depletion of RANTES^−/−^ mice could further increase the severity of exhaustion. When we compared CD8 T cell cytokine production, we found that the reduced IFNγ production in RANTES^−/−^ mice was similar to that of WT mice depleted of CD4 T cells. Moreover, the depletion of CD4 T cells in RANTES^−/−^ mice did not further decrease cytokine production (**figure 11A**). CD4-depletion of WT and RANTES^−/−^ mice ablated any difference in cytokine potential of CD8 T cells (**figure 11D**) and resulted in similarly high viral titers in WT and RANTES^−/−^ mice (**figure 11E**). This observation suggested that RANTES plays a role in mitigating the severity of exhaustion and that either RANTES^−/−^ CD4 T cells provide little benefit to the CD8 T cell response in RANTES^−/−^ mice or the higher viral load in RANTES^−/−^ mice drives more severe CD8 T cell exhaustion despite the CD4 T cells.

Transcription factors have recently been demonstrated to play a key role in regulating CD8 T cell exhaustion during clone 13 infection. We have recently found that Tbet is downregulated in exhausted CD8^+^ T cells during chronic LCMV infection and this downregulation is accentuated in the absence of CD4 help (Kao et al. submitted) (**figure 11F**). This loss of Tbet results in more severe T cell exhaustion during chronic viral infection. We therefore next examined whether Tbet expression was impacted by the loss of RANTES. In chronically infected RANTES^−/−^ mice Tbet expression was substantially lower than in WT mice (**figure 11F**). In fact, the loss of RANTES alone reduced Tbet expression in virus-specific CD8 T cells to levels seen in CD4 depleted WT mice. To determine whether this loss of Tbet was also seen earlier during clone 13 infection, we examined Tbet expression at day 8 p.i. Tbet expression was already slightly reduced by day 8 p.i. (this reduction reached significance with the D^b^GP276-specific CD8 T cells but only a trend in the D^b^GP33-specific CD8 T cells) (**figure 11G**). Reduced Tbet expression was consistent with the reduction in IFNγ production observed at this early time p.i.

## Discussion

The role of chemokines in regulating immune responses during chronic viral infections is poorly understood. Here we investigated the importance of RANTES in response to a chronic infection where CCR5 is not a viral co-receptor. RANTES was more highly expressed during chronic LCMV infection compared to acute infection. While the absence of RANTES did not impact T cell responses following acute LCMV infection, a different scenario emerged during chronic LCMV infection. During chronic infection, CD8 T cells become exhausted and their dysfunction was characterized by a loss of cytokine production, reduced cytotoxicity and increased inhibitory receptor expression, all of which can hinder the ability to control the infection [31], [32], [43], [44]. In the absence of RANTES, CD8 T cell exhaustion was more severe with reduced virus-specific CD8 T cell numbers, cytokine production and higher expression of inhibitory receptors. The cytotoxic potential of virus-specific CD8 T cells responding to clone 13 infection in RANTES^−/−^ mice was also reduced compared to WT controls. Consistent with the more severe exhaustion of the CD8 T cell response, mice lacking RANTES also had higher viral loads. Thus, the absence of RANTES resulted in the dysfunction of virus-specific CD8 T cells and poor viral control suggesting that RANTES has an important role in regulating and/or sustaining optimal immune responses during chronic viral infection.

There are a number of ways in which the absence of RANTES could result in the higher viral titers and reduced CD8 T cell function during clone 13 infection. First, slightly higher viral loads at the beginning of the response could lead to more severe CD8 T cell exhaustion. One possible mechanism for RANTES affecting viral load is via one of the main cell types infected by LCMV, macrophages. Macrophages play a key role in the immune defense against LCMV. Marginal zone macrophages and metallophilic macrophages may act as filters, controlling the spread of LCMV [45]. The increased tropism of LCMV clone 13 for macrophages and DCs is thought to result in the ability of the virus to persist [46]. The absence of RANTES could impact macrophage function or survival. For example, RANTES is essential to prevent apoptosis of macrophages infected with Sendai virus [19]. Thus, it will be important to investigate the role of RANTES in regulating DC and macrophage differentiation during persisting infections. A second possibility is that RANTES regulates the homing dynamics of the T cells, preventing T cell migration to the peripheral tissues or microenvironments and therefore limiting the ability of these cells to control the infection. However, during chronic LCMV infection the LCMV-specific CD8 T cells were found in spleen, blood and liver showing that the virus-specific T cells could still migrate to peripheral tissues at least at the level of the whole tissue. This observation does not rule out potential differences in movement within tissue, however, and a more detailed analysis of the migration dynamics of exhausted CD8 T cells in the absence of RANTES could be important. Third, CD4 T cell help could be reduced/absent in mice lacking RANTES. At least with LCMV Armstrong infection, CD4 T cell help appears to be intact as CD8 T cell memory cells are fully functional upon secondary challenge. Moreover, LCMV-specific CD4 T cell expansion and cytokine production in RANTES^−/−^ mice were similar to WT mice in response to both LCMV Armstrong and LCMV clone 13. While the phenotype of the CD8 T cells in RANTES^−/−^ mice was similar to CD4-depleted mice, the viral titers in mice lacking CD4 T cells was much higher suggesting that the CD4-depleted phenotype is more severe. Given that CD4 T cells also produce RANTES, it is possible that CD4 T cells are an important source of RANTES during LCMV clone 13 infection but that remains to be determined. A fourth possibility is that RANTES directly affects T cell activation/differentiation leading to reduced effector functions and that loss of RANTES directly results in functional defects in T cells leading to higher viral loads. While RANTES has been shown to act as a costimulator of T cells [47], [48], CCR5^−/−^ T cells responded similarly to WT CD8 T cells in a competitive environment suggesting that the importance of RANTES during LCMV clone 13 infection was not due to direct costimulation of CD8 T cells, though other receptors capable of binding RANTES could have a role.

CD8 T cell activation/differentiation is clearly negatively impacted by the absence of RANTES since IFNγ production by CD8 T cells was reduced even at day 8 p.i. in RANTES^−/−^ mice and this reduced IFNγ production was even more dramatic at the chronic stage of disease. Given that IFNγ has been shown to regulate the ability to clear LCMV infection [44], [49], [50], this initial decrease in IFNγ at the early stage of infection could result in a reduced ability to control viral replication, leading to further CD8 T cell exhaustion. Our data supports a role for RANTES in allowing the efficient activation and differentiation of CD8 T cells that are required to help control clone 13 infection. Interestingly, RANTES was not required for memory CD8 T cells to clear clone 13 infection. This observation, along with the similar T cell response to acute LCMV infection supports a role for RANTES during a sustained infection and further supports the model that minor defects early in the response to a rapidly disseminating infection are magnified as the infection persists leading to more severe T cell dysfunction and pathogen persistence.

Transcription factors that regulate effector functions of CD8 T cells during LCMV infection include Tbet and eomesodermin [51], [52]. Tbet expression was reduced in the absence of RANTES during LCMV clone 13 infection. How the absence of RANTES regulates the expression of Tbet, however, is currently unclear. These findings do suggest that the CD8 T cells responding to clone 13 in RANTES^−/−^ mice have differential expression of transcription factors compared to those from WT mice and perhaps these differences in transcription factor regulation impact their effector functions. Determining whether this effect can be directly attributed to RANTES or is a byproduct of higher viral load requires further investigation.

Interestingly, while CD8 T cell numbers and function were clearly reduced in the absence of RANTES, the CD4 T cells were not as sensitive to the loss of RANTES. CD4 T cells were unaffected in terms of numbers and the ability to produce IFNγ. Thus, the absence of RANTES had differential effects on CD4 versus CD8 T cells. These observations are somewhat surprising given that both CD4 and CD8 T cells produce RANTES and express the main receptor, CCR5. Further, this observation suggests that at least some aspects of CD4 and CD8 T cell exhaustion are regulated differently during chronic LCMV infection. Perhaps the differential effects of RANTES on CD4 versus CD8 T cells could be due to differences in expression of the other receptors for RANTES.

The CCR5-Δ32 mutation is found at a high frequency in European populations and is thought to have arisen through selective pressure during Yersinia pestis or variola major infection [53]. While absence of CCR5 can clearly be protective against HIV, CCR5 plays a role in protecting against WNV and tick-borne encephalitis. CCR5 may also play a protective role in the response against yellow fever virus; viscerotropic disease following yellow fever virus (YFV) vaccination in one subject was associated with the CCR5-Δ32 polymorphism as well as an additional mutation in the RANTES promoter [54]. The dichotomy of protection versus susceptibility of various infections and the use of CCR5 inhibitors suggests the need for more research on subjects with the CCR5-Δ32 mutation in terms of susceptibility to infection with different pathogens.

Understanding the role of RANTES during chronic infection is highly relevant due to the interest in CCR5 inhibitors for the treatment of HIV. CCR5 inhibitors prevent the entry of the R5-tropic stains of HIV virus into the cell [26]. While CCR5 inhibitors can be of tremendous benefit to those infected with the CCR5-tropic stain of HIV, our data suggests that blocking the RANTES pathway could negatively influence ongoing immune responses to other persisting infections. Many patients infected with HIV are also co-infected with other pathogens and the effect of the RANTES∶CCR5 pathway on these co-infections is not well understood. As many as 30% of HIV-infected patients in western Europe and the USA are coinfected with hepatitis C virus (HCV) and complications from HCV coinfection have emerged as a significant cause of morbidity and mortality [55], [56], [57]. Given the role of RANTES in regulating responses to the flaviviruses WNV and YFV, and that serum levels of CC-chemokines are increased in patients infected with chronic hepatitis [58], it will be interesting to determine whether RANTES also plays a role in regulating T cell responses to another member of the flavivirus family HCV.

Our data suggest that therapeutic interventions targeting the RANTES pathway could have negative effects on the ability to control some chronic infections and indicates the need for further research into any link between the CCR5-Δ32 mutation and persistent infections. These observations also suggest that blocking the RANTES∶CCR5 receptor pathway could alter the development and or quality of antiviral immune responses to chronic viral infection and, therefore, CCR5 inhibitors that block only HIV binding but not the RANTES∶CCR5 pathway may be more ideal.

## Supporting Information

Figure S1 **RANTES deficient P14 CD8 T cells respond similarly to WT P14 cells in a WT environment.** WT and RANTES^−/−^ P14 Tg T cells were transferred into C57BL/6 mice and the mice infected with LCMV clone 13 the following day (**A**). The gating strategy for identifying WT and RANTES^−/−^ P14 cells is shown (**B**). On days 10–14 p.i., the WT versus RANTES^−/−^ P14 cells were examined for expression of PD-1 (**C**) and also cytokine production after stimulation with GP33-44 peptide (**D and E**). Representative FACs plots are shown (**D**) and MFI of IFNγ as well as the percentage of IFNγ producing cells also making TNFα (**E**).
(EPS)
